# Binding Capacity and Adsorption Stability of Uremic Metabolites to Albumin-Modified Magnetic Nanoparticles

**DOI:** 10.3390/ijms26115366

**Published:** 2025-06-03

**Authors:** Indu Sharma, Agatha Milley, Lun Zhang, Jiamin Zheng, Ethan Lockwood, David S. Wishart, Marcello Tonelli, Larry D. Unsworth

**Affiliations:** 1 Department of Chemical and Materials Engineering, University of Alberta, Edmonton, AB T6G 2R3, Canada; indu2@ualberta.ca (I.S.); amilley@ualberta.ca (A.M.); elockwoo@ualberta.ca (E.L.); 2 The Metabolomics Innovation Centre, University of Alberta, Edmonton, AB T6G 2E9, Canada; lun2@ualberta.ca (L.Z.); jiamin3@ualberta.ca (J.Z.); dwishart@ualberta.ca (D.S.W.); 3 Department of Biological Sciences, University of Alberta, Edmonton, AB T6G 2R3, Canada; 4 Department of Medicine, Cumming School of Medicine, University of Calgary, Calgary, AB T2N 2T8, Canada; cello@ucalgary.ca; 5 Department of Biomedical Engineering, University of Alberta, Edmonton, AB T6G 2R3, Canada

**Keywords:** iron oxide magnetic nanoparticles, albumin, hemodialysis, LCMS, PBUTs

## Abstract

Kidney disease causes the retention of uremic metabolites in blood, which is associated with many comorbidities. Hemodialysis does not properly clear many metabolites, including large, middle-sized, and small protein-bound uremic toxins (PBUTs). Adsorption strategies for metabolite removal require the development of engineered adsorbents with tailored surfaces to increase the binding of desired metabolites. Albumin is uniquely positioned for modifying blood-contacting surfaces to absorb uremic metabolites, as it (i) minimizes non-specific protein adsorption and (ii) binds a range of molecules at Sudlow Sites I and II with different affinities. It is unknown if albumin-modified surfaces retain the adsorption qualities of solution-free albumin, namely, adsorption stability or specificity. Herein, albumin was covalently attached to iron oxide nanoparticles and characterized using multiple methods. Metabolite adsorption was conducted by incubating particles in a model solution of thirty-three uremic metabolites associated with kidney failure. Adsorption efficiency, selectivity, and stability were affected by albumin concentration and incubation time. Metabolite adsorption was found to change with time, and it was more effective on albumin-modified particles than unmodified controls. The findings outlined in this paper are crucial for the design of next-generation advanced blood-contacting materials to enhance dialysis and blood purification for patients with kidney disease.

## 1. Introduction

Chronic kidney disease (CKD) affects 8 to 16% of the global population and ranks among the top 10 causes of death worldwide [1,2]. As kidney dysfunction progresses, the unavoidable accumulation of uremic metabolites (UMs) occurs [3,4]. Although life-saving, current hemodialysis (HD) methods fail to effectively remove all UMs, particularly protein-bound uremic toxins (PBUTs) and those with large distribution volumes, which remain at high concentrations due to reabsorption or recycling [4,5]. This has been linked to diminished life expectancy, reduced quality of life, significant comorbidity burdens, and high healthcare costs [6,7,8]. Given the constraints of hemodialysis and the minimal advancements in the lives of patients, it is estimated that kidney disease will rank as the fifth largest cause of death worldwide by 2040, so there is an urgent necessity for new effective treatment modalities [9,10].

Emerging adsorbent-based strategies have the potential to be used in wearable devices with many ideal attributes, like continual clearance, less water waste, less plastic waste, and better patient quality of life and health outcomes [11,12]. Magnetic nanoparticles (MNPs) have many notable properties for adsorbing metabolites from the blood, including a high surface area that is easily modified, proven biocompatibility, benign biodegradability, can be captured using exogenous magnetic fields, and low cost [13,14,15,16]. Blood-contacting adsorbents must be hemocompatible whilst having a sustained and specific binding of metabolites [17]. Albumin has been shown to bind a variety of molecules, including UMs, through Sudlow Sites I and II [18,19,20]. It is thought that modifying MNPs with albumin will enhance MNP hemocompatibility, dispersion stability, and oxidation resistance while providing a matrix to adsorb UMs [16,21,22,23,24].

In general, a significant gap exists in the literature on the adsorption of UMs from complex solutions of metabolites, where most work primarily focuses on individual toxins in single buffer solutions, and limited studies have addressed the uptake of these toxins by albumin-modified surfaces [25]. However, a plethora of metabolites accumulate in kidney failure patients that are linked with poor patient outcomes: xanthine, kynurenine, putrescine, creatinine, quinolinic acid, and polyamines [26,27,28,29]. Our lab has modified MNPs with (methacryloyloxy)ethyl phosphorylcholine-co-β-cyclodextrin (p(MPC-co-PMβCD)) films that are particularly effective in adsorbing UMs, displaying notable selectivity and time-dependent interactions [30]. Although MPC is considered a hemocompatible coating, it is possible that albumin modification will provide an increased density of metabolite binding sites and enhanced hemocompatibility through a simple surface modification approach.

UM interactions with albumin films are underexplored. Building upon our previous work on albumin-modified magnetic nanoparticles that studied their hemocompatibility [24], albumin-modified MNPs formed using similar techniques were exposed to a model solution of 33 UMs (Table 1) to understand how surface properties and incubation time affect metabolite binding capacity, adsorption changes with time, and adsorption specificity. Albumin coupling to an amino-silane-modified MNP was conducted at two different albumin solution concentrations (0.2 and 2 mg/mL), and formed films were characterized using thermogravimetric analysis (TGA), X-ray photoelectron spectroscopy (XPS), and zeta potential. Changes in the secondary structure of albumin within the coating were characterized using Fourier-transform infrared spectroscopy (FTIR), particle size, and morphology, and film thickness was quantified using transmission electron microscopy (TEM). Quantitative mass spectroscopic analysis was used to determine the adsorbed uremic toxin profile. Surface properties and incubation time (from 1 h to 4 h) were major determinants of metabolite adsorption—adsorption was not constant with time and not purely driven by solution concentration. These findings are pivotal for developing advanced, next-generation materials that enhance dialysis and blood purification, offering personalized treatment options for kidney failure patients.

## 2. Results

### 2.1. Particle and Film Dimensions

**Figure 1:**
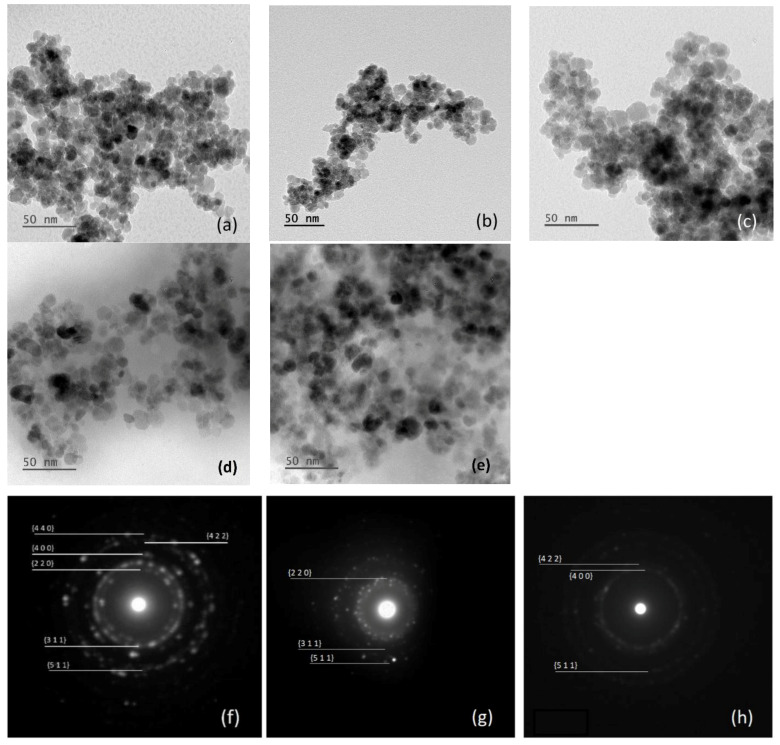
Representative TEM images for (**a**) bare nanoparticles, (**b**) APTES modified, (**c**) GA modified, (**d**) BSA (0.2) modified, and (**e**) BSA (2) modified, and SAED images of (**f**) bare nanoparticles, (**g**) BSA (0.2) modified, and (**h**) BSA (2) modified.

**Figure 2:**
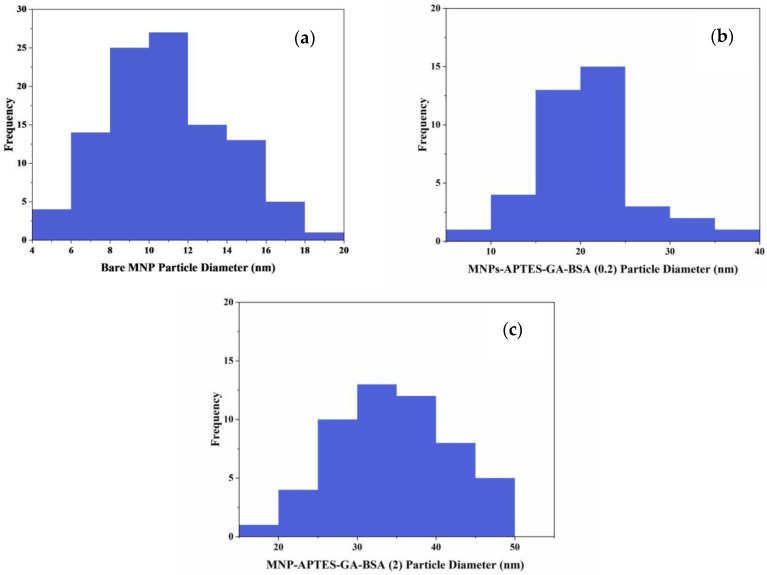
Particle size distribution of (**a**) bare nanoparticles (n = 104), (**b**) BSA (0.2) modified (n = 46), and (**c**) BSA (2) modified (n = 53).

MNPs were analyzed using TEM to determine their size, shape, and homogeneity, both before and after albumin modification (Figure 1). Bare MNPs were mostly uniform and spherical, with diameters ranging from 8 to 14 nm, averaging 10.6 ± 3.1 nm (n = 104; Figure 2a). Modifications with APTES (NH_2_(CH_2_)_3_Si(OC_2_H_5_)_3_) and glutaraldehyde showed minimal size or shape changes, consistent with previous studies (Figure 1b,c) [40]. The TEM images showed the formation of an albumin layer (appearing as a lighter outline around the particles) in Figure 1d,e. The average diameters of particles coated with albumin were 20.9 ± 5.2 nm at 0.2 mg/mL and 34.7 ± 3.2 nm at 2.0 mg/mL albumin concentrations (Figure 2b,c). The 0.2 mg/mL result was consistent with a thin albumin layer, as expected from the hydrodynamic radius of bovine serum albumin (BSA) [41]. At 2 mg/mL, particle sizes ranged from 27 to 42 nm, varying with the nanoparticle type and albumin attachment method, as previously reported [21,40,41]. Higher concentrations of albumin led to MNPs with a globular, uniformly spherical shape and the formation of larger aggregates. There was also some excess protein, indicated by a light, cloudy residue on the TEM images, which obscured the precise measurement of the albumin coating thickness, though a substantial, densely coupled protein layer was present. Origin 2024’s *t*-test was used to conduct statistical analysis. Calculated *p*-values for bare and MNPs-APTES-GA-BSA (0.2), bare and MNPs-APTES-GA-BSA (2) and MNPs-APTES-GA-BSA (0.2), and MNPs-APTES-GA-BSA (2) were *p* < 0.0001, *p* < 0.0001, and *p* < 0.001, respectively.

Selected area electron diffraction (SAED) analysis was performed on bare MNPs, MNPs-APTES-GA-BSA (0.2), and MNPs-APTES-GA-BSA (2) (Figure 1f–h). The results were consistent with the structure of magnetite, with the SAED images indicating the presence of the {311}, {400}, {422}, {511}, and {440} families of planes. The diffraction analysis of Alb-MNPs showed similar patterns to bare particles—more dots were visible due to the protein layer. SAED analysis could not be properly conducted for thicker albumin layers, as the amorphous protein interfered with the diffraction pattern.

### 2.2. Relative Mass of Alb-MNP Films

**Figure 3:**
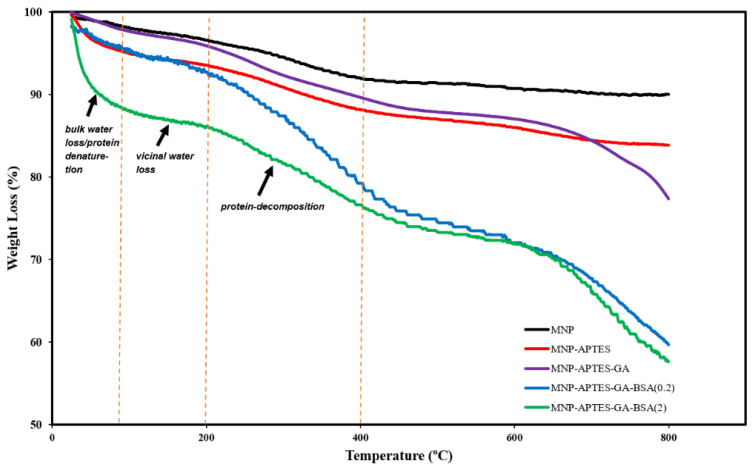
Thermogravimetric analysis for bare, subsequently added APTES, GA, BSA (0.2), and BSA (2) nanoparticles.

For each modification step, TGA was conducted and summarized for all types of nanoparticles (Figure 3). The mass reduction observed below 100 °C was due to the evaporation of bulk water, while that between 100 and 200 °C was due to vicinal water content, which was likely trapped within the particle matrix. Bare nanoparticles exhibited approximately 9.5% weight loss, signifying minimal water content and absence of deterioration [6]. Validation of every modification was confirmed by additional mass loss in every step. Upon addition of glutaraldehyde, the reduced mass loss at lower temperatures and increased mass loss at higher temperatures indicated enhanced thermal stability, which is in agreement with our previous studies on similar systems—illustrating consistent surface modification [24].

The deterioration of BSA in films was not influenced by the BSA concentration. Further, up to 100 °C mass loss was attributed to denaturation of BSA, and mass loss before 200 °C was attributed to water loss [24,42]. Furthermore, albumin decomposed in the temperature range of 250–400 °C. Up to 110 °C, a broad peak was observed, where an endothermic effect has been attributed to BSA denaturation [5,24,42]. A noticeable change occurred between 180 and 500 °C, which is related to the decomposition of BSA, glutaraldehyde, and amine functional group complex. We propose that the noticeable shift in the maximum decomposition rate with varying BSA concentration confirms the adsorption of BSA on the magnetic nanoparticles [42]. Upon increasing the BSA concentration (0.2 to 2 mg/mL), the peak intensity increased, which reflected the shift toward higher mass loss. Nanoparticles modified with a high BSA concentration (2 mg/mL) showed the highest mass loss between 200 and 380 °C, which was linked to C-O and C-C bond breakdown, confirming the high amount of BSA coating, which can be confirmed by TEM images.

### 2.3. Surface Charge Analysis

**Figure 4:**
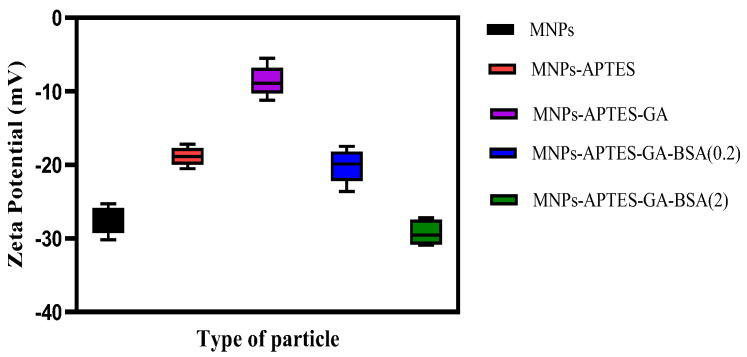
ζ-potential for bare and subsequently added APTES, GA, BSA (0.2), and BSA (2) nanoparticles.

Zeta potential measurement revealed the significant variation on surface modification of MNPs. Bare, APTES, APTES-GA, APTES-GA-BSA (0.2), and APTES-GA-BSA (2) MNP systems exhibited zeta potentials of −27.6 ± 2, −18.8 ± 1, −8.5 ± 2, −20.16 ± 2, and −29.25 ± 2 mV, respectively (Figure 4), at pH 7, consistent with previously reported values on similar surfaces [21,24,43,44]. The successful modifications with APTES and glutaraldehyde were further confirmed thorough subsequent changes in surface charge. It was observed that the addition of albumin significantly reduced the zeta potential due to its negative surface charge at this solution pH [43]. An increased concentration of albumin solution led to a greater negative surface charge, which has been correlated with improved suspension stability [45].

### 2.4. Film Bonding Chemistry

**Figure 5:**
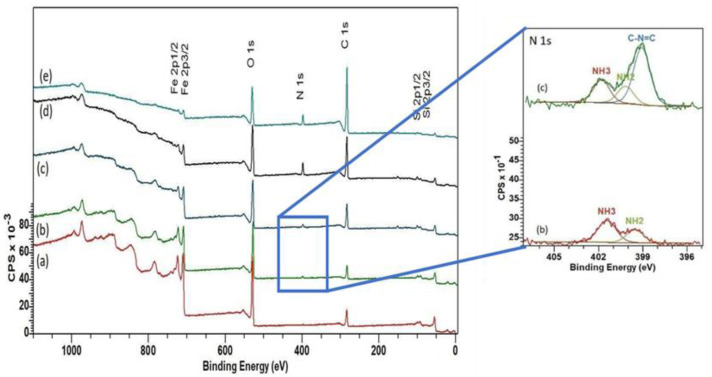
XPS scan for bare (**a**) and subsequently added APTES (**b**), GA (**c**), BSA (0.2) (**d**), and BSA (2) (**e**) nanoparticles. (Inset) N 1s, high-resolution scans for APTES- and GA-modified nanoparticles. Accurate fit determined using χ^2^ and full width at half maximum of component peaks.

An XPS survey scan of MNPs before and after modification was conducted (Figure 5). Previous studies indicated the absence of a satellite peak between Fe 2p1/2 and Fe 2p3/2 for Fe_3_O_4_, and our current investigation supported this finding [24,46,47]. The peak positions for Fe 2p3/2 and Fe 2p1/2 were at 710.6 and 724.1 eV, respectively. The peaks observed for Fe 3p3/2 at approximately 55 eV, O(1s) at 529.5 eV, and C (1s) at 283.5eV corresponded to the Fe-O bond characteristic of the Fe_3_O_4_ nanoparticles (as expected) [47]. The presence of nitrogen and silica peaks in the survey scan confirmed silanization. The N 1s peak in APTES and its modified surfaces formed from the convergence of two separate peaks of NH_2_ at 400.7 eV and NH_3_^+^ at 401.8 eV, and the Si 2p peak at 98.7 eV. Similar to previous literature, the elevated protonation level in the amine groups at the end of the APTES chain was confirmed by the high-resolution scan of the nitrogen peak (Figure 5, inset) [48]. A noticeable shift in the N 1s peak following the addition of glutaraldehyde indicated significant chemical modification and the formation of new bonds: C-N = C found at 399.05 eV assigned to the aldehyde group in glutaraldehyde and the amine group in APTES [49].

The successful film coating was further confirmed by the intensity shift in the O 1s, C 1s, and Fe 2p peaks. Initially, the major contribution to the peak, observed at 529.5 eV, originated from oxygen in the crystal structure, typical of oxide crystal formations. A hydroxyl functional group on the nanoparticle surface was also identified at 530.9 eV. Subsequent APTES modification shifted the hydroxyl peak to 531.6 eV due to covalent bonding with silicon on the surface. The N 1s peak increased with APTES, and the N 1s and C 1s peaks increased with albumin, suggesting the covering of the Fe surface and increased fraction of these components within the escape distance of the photoelectron. Moreover, albumin binding to the magnetic nanoparticle led to significant peak shifts attributed to various functional groups within the protein structure, including OH, O-C, O-H, O-N, and O-C-N.

XPS analysis was used to determine the chemical composition of samples (Table 2). The detection of nitrogen and silicon values validated the effective surface modification achieved by APTES. The values for nitrogen and silicon were very similar before adding albumin, which was expected, as APTES contains a 1:1 ratio of nitrogen and silicon, and glutaraldehyde includes neither. The atom % of iron decreased with each chemical addition, which was expected, as the coating thickness increased. With the addition of albumin from 0.2 to 2 mg/mL, the relative carbon content in the samples significantly increased for higher concentrations of BSA film, indicating a densely packed albumin layer. Significant carbonaceous contamination was present in the bare MNP sample. These values were similar to those previously reported for surface modification by APTES and GA on MNPs [48,49].

## 3. Discussion

### 3.1. Effect of Film Formation on Albumin Secondary Structure

**Figure 6:**
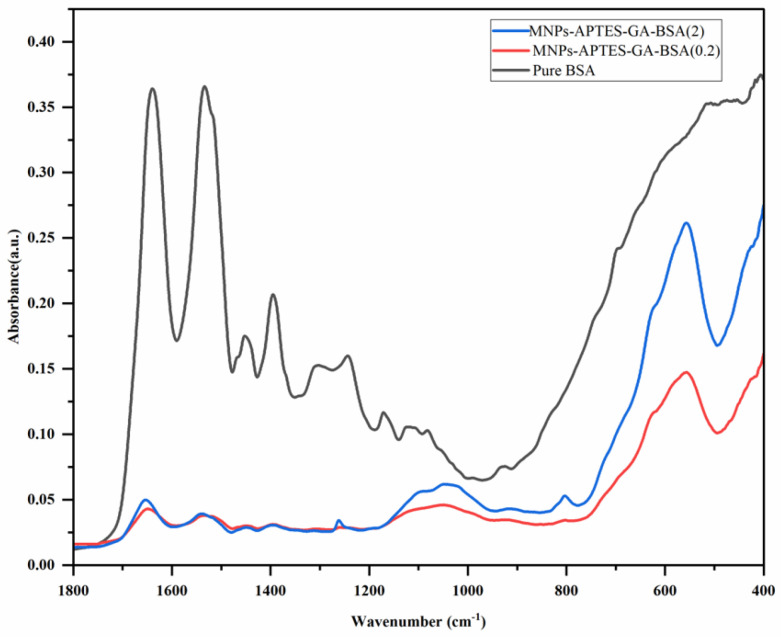
FTIR scan of pure BSA, MNPs-APTES-GA-BSA (0.2), and MNPs-APTES-GA-BSA (2).

**Figure 7:**
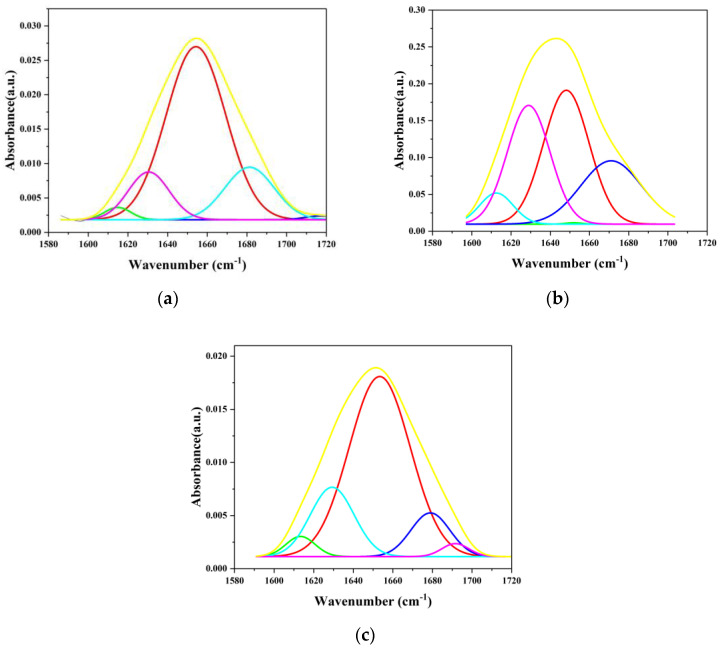
FTIR Gaussian fitting of amide-I band for (**a**) pure albumin, (**b**) MNPs-APTES-GA-BSA (0.2), and (**c**) MNPs-APTES-GA-BSA (2), respectively.

Fourier-transform infrared spectroscopy (FTIR) was utilized to observe changes in the secondary structure of albumin to understand the extent of changes upon incorporation within the film. This was done to understand if denaturation was so extensive that the Sudlow Sites could not be maintained, thus altering UMs’ retention. It was confirmed that albumin was present on the MNP surface (Figure 6), as indicated by the characteristic amide-I (-NH_2_-) stretching peaks at 1649, 1648, and 1653 cm^−1^, as well as the amide-II (-NH-) bending peaks at 1533, 1539, and 1534 cm^−1^. The drop in intensity suggested a structural transition from α-helix to β-sheets and dehydrated β-turns, confirmed by the 1394, 1385, and 1396 cm^−1^ peaks. Alterations in dehydrated β-turns were identified using the bands at 1243, 1248, and 1246 cm^−1^. The presence of the Fe-O bond was also confirmed using the peaks at 556 and 634 cm^−1^. The percentage of secondary structural elements was quantified by fitting the amide-I band region with Gaussian curves and finding the multi-peak position using Fourier self-deconvolution (FSD) of the protein amide-I band. Multicomponent peak areas were quantified using Gaussian fitting in Origin 2024 (Figure 7).

Characterization of the secondary structures of BSA was conducted (Table 3) using the amide-I region, as determined using: (i) dehydrated β-turns, 1694 cm^−1^; (ii) β-sheet/turns, 1685–1663 cm^−1^; (iii) α-helix, 1660–1650 cm^−1^; (iv) random coil, 1648–1638 cm^−1^; (v) β-sheet, 1639–1621 cm^−1^; (vi) side chain, 1616–1600 cm^−1^ [50,51]. A variability of ~10% in secondary structures has been previously reported, with an α-helix content typically comprising 58–65% of the total secondary structure [52,53,54]. The α-helix content is essential, as Sudlow Sites I and II reside in helix-rich protein regions. The α-helix content for BSA has been shown to decrease upon film formation, and random coil content increased compared to when in solution [53,54]. Loss of helical structure was greater for MNPs-APTES-GA-BSA (0.2) than MNPs-APTES-GA-BSA (2), possibly due to the covalent bonding of albumin at the surface. Previous studies have indicated that the loss of α-helix structure was compensated by the emergence of beta structures due to the relative stability of beta structures compared to α-helices [50,53]. For MNPs-APTES-GA-BSA (2), the loss of β-sheets was more than that for MNPs-APTES-GA-BSA (0.2), while the major gain for MNPs-APTES-GA-BSA (2) was in β-turn, and for MNPs-APTES-GA-BSA (0.2) was in the random chain and dehydrated β-turns. This indicated that the albumin film was destabilized due to interaction with the MNP surface. However, the β-sheets remained low, indicating that no BSA aggregation occurred for MNPs-APTES-GA-BSA (0.2) [51,55]. Additionally, for MNPs-APTES-GA-BSA (2), the increased content of β-turns may prevent denaturation by inhibiting the interaction that exposes protein fragments susceptible to major conformational changes that lead to the unfolding of the protein [56]. It seems that changes in α-helix content transitioned to increased random coils and dehydrated β-turns for the MNP-APTES-GA-BSA (0.2) system, suggesting a hard protein corona formation with slight random coil formation. The higher-concentration system showed no dehydrated β-turn formation, suggesting a soft corona formation and only a moderate increase in β-sheet and β-turn composition. It seems that tethered albumin underwent a change in protein structure, but the protein remained intact and similar to its native form [52,57].

### 3.2. Uremic Metabolite Adsorbed Amounts

**Figure 8:**
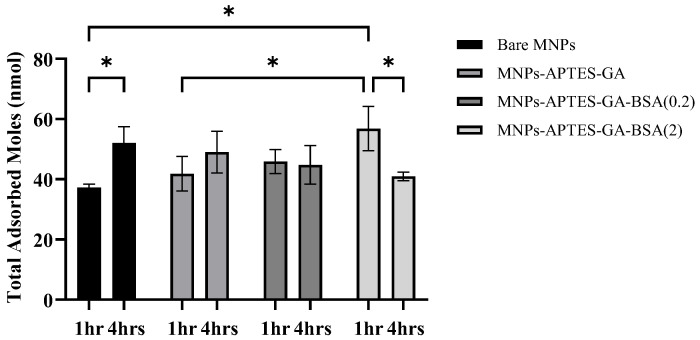
Total adsorbed metabolites for all MNP systems after 1 or 4 h of incubation in the model UM solution. Asterisks denote statistical significance between samples, with * representing *p* < 0.05. Data represent the average ± 1 STD, n = 3.

It was observed that all MNP systems adsorbed metabolites (Figure 8), but the quantity and types of metabolites were dependent upon the MNP surface and incubation time. The total adsorbed UMs for Alb-MNPs (0.2 mg/mL) were ~45 and ~44 nmol at 1 and 4 h of incubation, respectively. Alb-MNPs formed from 2 mg/mL films showed the highest adsorption at 1 h, followed by a decrease after 4 h from ~57 to ~41 nmol. For 1 and 4 h of incubation on bare MNPs and MNP-APTES-GA particles, the total adsorbed mols were ~37 to ~51 nmol and ~40 to ~49 nmol, respectively. In the case of bare particles (1 and 4 h) and MNPs-APTES-GA (1 and 4 h), an increase in incubation time yielded an average increase in total mols of adsorbed metabolites. On average, the total mols adsorbed decreased with the increasing incubation time for MNPs-APTES-GA-BSA (2), suggesting metabolites may be displaced with time in this system. That said, in all ANOVA tests using Tukey’s multiple comparison tests, the *p*-value for bare MNPs (1 h) vs. bare MNPs (4 h) was 0.046, bare MNPs (1 h) vs. MNPs-APTES-GA-BSA (2) for 1 h was 0.013, MNPs-APTES-GA (1 h) vs. MNPs-APTES-GA-BSA (2) for 1 h was 0.045, and MNPs-APTES-GA-BSA (2) for 1 h vs. MNPs-APTES-GA-BSA (2) for 4 h was 0.034, and others were not significantly different.

### 3.3. Uremic Metabolite Adsorption Selectivity

Adsorption selectivity was evaluated by normalizing the adsorbed UM (mol%) to that of the corresponding UM amount (mol%) in the original solution (Table 4). All MNPs had selectivity of 20 to 40 and 2 to 4 times greater than the original solution concentration for the neutral pyruvic acid and methylhistidine [58,59]. This was despite the low initial mol% of methylhistidine in the original solution. In contrast, none of the MNPs adsorbed detectable quantities of phenylacetic acid, even though it was 42 mol% of the initial solution. Similarly, L-asparagine, L-tyrosine, p-hydroxylphenylacetic acid, putrescine, homocysteine, xanthine, spermidine, spermine, argininic acid, and asymmetric dimethylarginine were not detected. Hippuric acid and creatinine, present in original solutions at 16 and 15 mol%, respectively, showed a significantly lower adsorbed mol%. An increased selectivity for 4-ethylphenyl sulfate and indoxyl sulfate was observed for most Alb-MNPs. Although, indoxyl sulfate and p-cresol sulfate binding with albumin was expected to be enhanced, while this was not observed for p-cresol sulfate under these conditions [19,20]. Both p-cresol sulfate and hippuric acid bind to Sudlow Site II, and their reduction may arise due to the denaturation of Sudlow Site II upon film formation or other compounds blocking access to this site [18,20]. Other than bare MNPs, the adsorption of indoxyl sulfate was up to 1.7 times that of the initial solution, increasing with the increased incubation time. These findings strongly demonstrated that individual metabolite adsorption was not solely dependent on their concentration in the incubation solution, as previously observed [30].

Uremic metabolite adsorption after 1 and 4 h of incubation was analyzed (Table 5) and showed that the interaction between metabolites and surfaces changed with time, a result considered holistically earlier in Figure 8 but now discussed on a per UM basis. Pyruvic acid was found to be highly adsorbed, which greatly affected the total adsorbed amounts of metabolites for each MNP system and the selectivity of the particles with time. Bare MNPs and MNP-APTES-GA particles exhibited a significant decrease in mol% for every compound except pyruvic acid and kynurenine, which showed an increase. Interestingly, MNP-APTES-GA-modified MNPs exhibited a decrease in mol% for most of the metabolites except for kynurenine, pyruvic acid, indoxyl sulfate, and 4-ethylphenyl sulfate, which had a substantial increase in mol% of 100, 18.6, 17.9, and 1.9, respectively, suggesting that this surface chemistry has a high selectivity for kynurenine, pyruvic acid, and indoxyl sulfate.

Conversely, Alb-MNPs had a significant increase in almost every compound except for pyruvic acid, 4-ethylphenyl sulfate, and kynurenine from 1 to 4 h of incubation. The latter showed drastic decreases of up to 420% between 1 and 4 h. This trend was magnified for all UMs adsorbed to MNP-APTES-GA-BSA (2 mg/mL) systems, suggesting that more albumin led to more binding than 0.2 mg/mL systems. These Alb-MNP results were directly opposite to the bare MNP and MNP-APTES-GA systems. Most notably, bare MNPs and MNP-APTES-GA showed a substantial increase in mol% of pyruvic acid from 1 to 4 h of incubation. In contrast, adding albumin yielded a significant decrease in the same timeframe, perhaps indicating that with increased time, pyruvic acid was being displaced from the surface. Interestingly, except for pyruvic acid and kynurenine, it was observed that albumin films formed at 2 mg/mL yielded increased amounts of all adsorbed UMs compared to their 0.2 mg/mL counterparts. Not only did the presence of albumin affect pyruvic acid stability, but a thicker film may have more binding sites available for these other metabolites, allowing for their increase in mol% of the total adsorbed species. Bare MNPs showed a complete decrease in adsorbed amount for all types of PBUTs (quinolinic acid > indoxyl sulfate > p-cresol sulfate > hippuric acid > indole acetic acid > kynurenine), while Alb-MNPs showed a moderate to substantial increase of these (except kynurenine) over this period. Most PBUTs have an aromatic ring and an ionic functional group, allowing many potential binding mechanisms, where enthalpy-driven interactions play a role [12,60,61]. Additionally, indoles possess a positive charge that may enable electrostatic interactions with negatively charged Alb-MNPs [61]. Finally, despite partial protein denaturation, Sudlow Sites I and II may be available for binding these metabolites. Ultimately, the high selectivity of Alb-MNPs for PBUTs may be attributed to multiple factors associated with the presence of albumin. Xanthosine and methylhistidine (both molecules are heterocyclic aromatic compounds) have seen a maximum mol% increase in the adsorbed amount for Alb-MNPs. Hence, we can state that Alb-MNPs not only adsorbed protein-bound uremic toxins but also strongly adsorbed non-PBUTs of middle- and low-molecular-weight molecules.

### 3.4. Uremic Metabolite Binding Characteristics of Albumin vs. Cyclodextrin Films

Previous work has investigated the use of cyclodextrins (CDs) on the adsorption of similar metabolites [30]. The adsorbed nmol of UMs to Alb-MNPs ranged from ~40 to 60 nmol. CD-modified MNPs adsorbed between ~20 and 180 nmols of total UMs for the same mass of particles. These data suggest that the number of binding sites available for metabolites on CD-modified MNPs was significantly larger than that of Alb-MNPs. After 1 h, type D (Film: 25% CD, 75% MPC) exhibited the highest UM (176.45 nmols) adsorption, while type C (Film: 50% CD, 50% MPC) showed the highest (152.58 nmols) adsorption after 4 h. Interestingly, types A (Film: 100% CD) and C demonstrated a significant increase in adsorbed metabolites over time from 16 to 152 nmols, while others showed a decrease from 176 to 35 nmols, though statistically significant changes were observed only for Types A, B (Film: 75% CD: 25% MPC), and D. Similarly, in this study at 1 h, the Alb-MNPs with 2 mg/mL showed the highest (57 nmols) adsorption, while at 4 h, bare MNP was higher (51 nmols). When surfaces had similar zeta potentials, both CD (Type D, −28.3 mV) and albumin (MNPs-APTES-GA-BSA (2), −29.25 mV) coatings showed similar trends, namely, highest adsorbed amounts after 1 h (~60 nmol) and a decrease after 4 h (~42 nmol).

Alb-MNPs yielded two times the concentration of pyruvic acid and increased methylhistidine and creatinine compared to CD-MNPs, where neither were observed. Hippuric acid was concentrated by Alb-MNPs at ~2 to 5 times that of CD-MNPs. It was observed that indole-3-acetic acid did not adsorb to CD films, while its adsorption increased on all Alb-MNPs with time. Likewise, CD-MNPs showed significant spermidine and xanthine, whereas Alb-MNPs adsorbed none. Neither of these surfaces showed the adsorption of alginic acid, asymmetric dimethylarginine, homocysteine, phenolic acid, dimethyl glycine, and spermine.

Based on the heat map comparison presented in Table 4, Alb-modified MNPs have a more comprehensive range of adsorption selectivity from a complex 33 metabolite solution compared to CD-MNPs, which showed only 19 metabolites. While CD-MNPs seemed to have higher adsorption selectivity for only a few compounds (i.e., ethylphenyl sulfate, guanidinopropionic acid, pyruvic acid, and spermidine), Alb-MNPs showed higher adsorption selectivity for a broader range of compounds. These were, in decreasing order, pyruvic acid, methylhistidine, indoxyl sulfate, 4-ethylphenyl sulfate, creatinine, hypoxanthine, quinolinic acid, indole acetic acid, orotic acid, hippuric acid, phenylalanine, uridine, p-hydroxyhippuric acid, trimethylamine N-oxide, uric acid, kynurenine, and guanidinopropionic acid.

**Figure 9:**
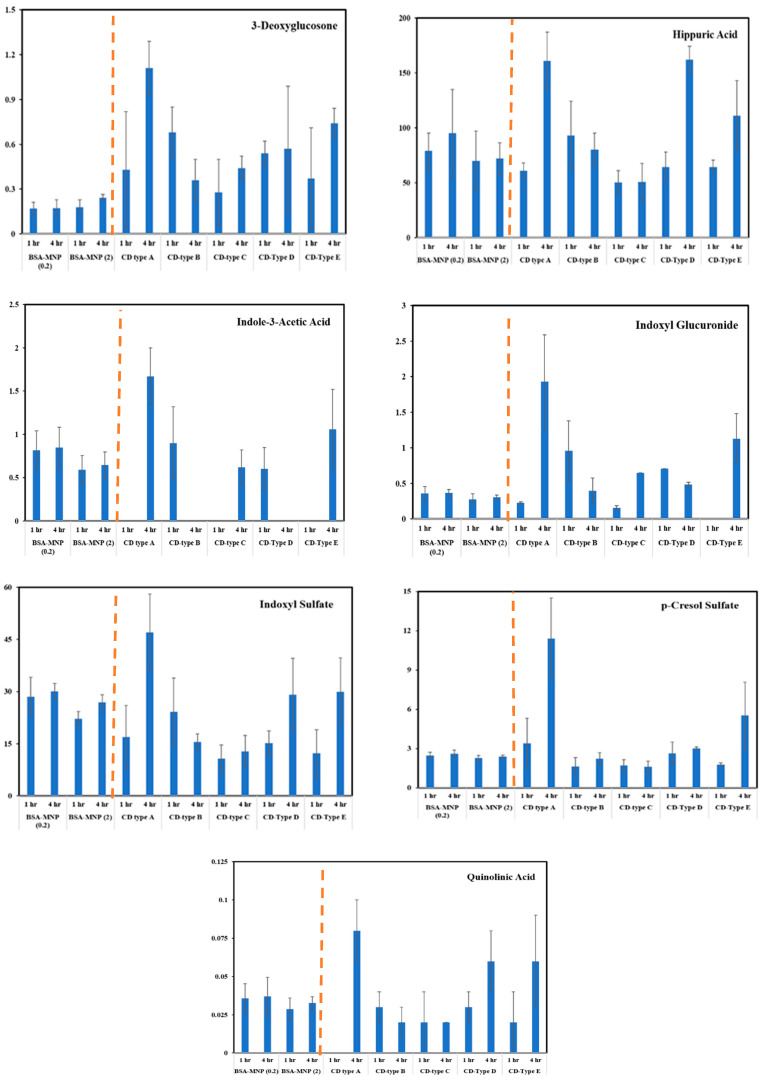
Quantitative LC/MS analysis of protein-bound uremic toxin (PBUT) adsorption on albumin-functionalized magnetic nanoparticles (Alb-MNPs; left of dashed line) and cyclodextrin-modified magnetic nanoparticles (CD-MNPs; right of dashed line). CD-MNPs (samples A–E) represent increasing molar ratios of cyclodextrin to MPC: A (1:0), B (3:1), C (1:1), D (1:3), and E (0:1). Adsorption profiles were assessed after 1 h and 4 h incubation. Data represent eluted metabolite concentrations (μM), expressed as mean ± standard deviation (n = 3). Blank values indicate concentrations below the detection limit of the method (<LOD).

Adsorption of 3-deoxyglucosone, hippuric acid, indole-3-acetic acid, indoxyl glucuronide, indoxyl sulfate, p-cresol sulfate, and quinolinic acid on CD- and Alb-modified films are summarized as eluted concentrations (µM) in Figure 9. Even though albumin may have a high binding affinity for PBUTs, the sheer number of binding sites afforded by a CD-rich film yielded increased amounts of adsorbed PBUTs. In most cases, the 100% CD film (particle A, 4 h) had between 2 and 3 times (i.e., 50–126 nmols) the number of metabolites adsorbed by albumin films. This was further reflected by decreased amounts of CDs in the particles labeled B–E, which showed reduced adsorbed amounts of these PBUTs compared to particle A. Interestingly, even pure MPC films (particle E, 4 h) yielded more adsorbed metabolites than albumin films. This suggests that even though albumin was a larger molecule than MPC and MPC-CD polymers, the adsorbed amounts of metabolites were less, likely because most of the albumin structure did not participate in PBUT adsorption.

## 4. Materials and Methods

### 4.1. Materials

Chemicals for synthesis: Sodium hydroxide (Fisher Scientific, Waltham, MA, USA), glutaraldehyde (70%, EM, Electron Microscopy Sciences, Hatfield, PA, USA), FeCl_2_·4H_2_O, FeCl_3_·6H_2_O, NH_4_OH (25%, Sigma Aldrich, St. Louis, MO, USA), NH_2_(CH_2_)_3_Si(OC_2_H_5_)_3_ (APTES 99%, Sigma Aldrich, St. Louis, MO, USA), BSA (98%, Sigma Aldrich, St. Louis, MO, USA), CH_3_CH_2_OH (99.5%, Fisher Scientific, Waltham, MA, USA), and phosphate-buffered saline (PBS) tablets (0.01 M, pH 7.4, HPLC-grade water, filtered at 0.22 µm; Fisher Scientific, Waltham, MA, USA).

Uremic Metabolites: The following chemicals were purchased from Sigma Aldrich: 3-deoxyglucosone (≥75%), 3-indoleacetic acid (≥98%), asymmetric dimethylarginine (≥98%), creatinine (≥98%), L-tyrosine, L-asparagine, dimethylglycine (≥99%), hippuric acid (≥98%), hypoxanthine (≥99%), indoxyl glucuronide (≥98%), indoxyl sulfate, guanidinopropionic acid (≥97.5%), kynurenine (≥98%), methylhistidine, orotic acid (≥98%), p-cresol sulfate (≥95%), phenylalanine (≥98%), pyruvic acid (≥98%), quinolinic acid (≥97%), spermidine (≥99%), spermine (≥97%), trimethylamine N-oxide (≥95%), argininic acid, uric acid (≥99%), uridine (≥99%), xanthine (≥99%), and xanthosine. The 4-ethylphenyl sulfate (98%) was obtained from Apexbio in Houston, TX, USA, and 4-hydroxyhippuric acid (≥98%) from Cayman Chemical, Michigan, MI, USA.

### 4.2. Synthesis of Iron Oxide Nanoparticles

Iron oxide magnetic nanoparticles were synthesized using the procedure described in detail in [24]. A nitrogen-purged three-necked flask containing FeCl_2_·4H_2_O and FeCl_3_·6H_2_O in degassed water was stirred at 75 °C. Ammonium hydroxide (25%) was added and stirring increased. After 1.5 h, the nanoparticles were washed, separated using a magnet, and vacuum dried.

### 4.3. Functionalization with APTES and GA

The functionalization of Fe_3_O_4_ nanoparticles (50 mg) was performed as described in [24]. Briefly, the nanoparticles were suspended and sonicated in 5 mL of ethanol, then 35 µL of APTES was added, stirred, and reacted under a nitrogen atmosphere overnight, washed, and suspended in PBS. Following the addition of glutaraldehyde (0.6 mL, 70%), the suspension was stirred for 3 h, washed again, and stored at 4 °C.

### 4.4. Formation of BSA-Functionalized MNPs

BSA functionalization of nanoparticles was carried out as per the detailed procedures in [24,41]. BSA (0.2 or 2 mg/mL) was dissolved in half the PBS volume, and MNPs (0.08 mg/mL) were sonicated in the other half. The solutions were mixed and shaken at 150 rpm for 2 h. Then, the particles were washed with PBS and stored at 4 °C.

### 4.5. Particle Characterization

TEM (JEM-ARM200CF S/TEM, JEOL, Houston, TX, USA) at 200 kV was used to determine the particle size and morphology. For TEM, 5 µL of diluted MNPs was placed on a carbon grid and dried overnight. TGA (Pyris 1 TGA, Perkin Elmer, Waltham, MA, USA) was used to assess thermal stability with a 10 °C/min ramp from 25 to 800 °C under nitrogen. Sample weights were 0.84 mg for bare nanoparticles, 1.31 mg for APTES modified, 5.36 mg for MNP-APTES-GA, 0.21 mg for MNP-APTES-GA-BSA (0.2), and 0.42 mg for MNP-APTES-GA-BSA (2). Zeta potential was measured with a Zetasizer (Malvern Instruments, Worcestershire, UK) using 25 µL of 1 mg/mL MNP suspension in 3 mL of DI water. Surface chemistry was analyzed using XPS (Kratos Ultra XPS Spectrometer, Manchester, UK) with 1 mg of freeze-dried sample, as per the previously published protocol [24].

FTIR spectroscopy (Thermo Scientific Nicolet™ iS50 FTIR Spectrometer, Madison, WI, USA) was used to investigate the secondary structure of albumin in both the solution and within the albumin film on the MNP surface. After protein immobilization, 1 mg of freeze-dried powder for all samples was studied. For native BSA, 2 mg/mL of BSA solution was used. The spectra for the solvent and sample were collected at the resolution of 4 cm^−1^ and 64 scan rates to enhance the signal-to-noise ratio. Each sample spectrum was processed using OMNIC 7.3 software for solvent spectra, baseline correction, and smoothness. The amide-I band (1600–1700 cm^−1^) characteristic of proteins and the carbonyl stretching were fitted to Gaussian curves, which is the best method to quantify the percentage of secondary structural elements. The protein secondary structure was determined using the Fourier self-deconvolution (FSD) method with OMNIC software. Subsequently, multicomponent peak areas were quantified using Gaussian fitting in Origin 2024 to determine β-sheet, random coil, α-helix, β-turn, and antiparallel β-sheets contents using wavenumber ranges 1614–1634, 1635–1648, 1650–1658, 1662–1678, and 1680–1691 cm^−1^, respectively.

### 4.6. Uremic Metabolite Solution

Stock solutions of each UM were made with LC-MS-grade water and combined with uric acid in its powdered form, which was added last. The total volume was adjusted using PBS to form a model uremic toxin solution in 10 mM of PBS at a pH of 7.4.

### 4.7. MNP Incubation with Uremic Toxin Solution

All tubes were washed with HPLC-grade methanol and vacuum-dried before use. Experimental samples were prepared by adding 0.25 mg of particles to 0.5 mL of UM solution, incubated at 10 rpm at 37 °C for 1 or 4 h, magnetically separated from the metabolite solution, and the solution was removed. These particles were suspended in 100 µL of HPLC-grade methanol and then put in a shaker at 37 °C and 80 rpm for 24 h for the desorption of adsorbed species. MNPs were magnetically separated from the methanol, and methanol samples were centrifuged at 2100 rcf for 20 min to determine whether any particles were still present in the sample. The metabolite–methanol samples were stored at −80 °C until mass spectroscopy could be conducted, and no volume loss was observed.

### 4.8. Analysis of Adsorbed Metabolites

Plasma samples were analyzed using a targeted MS-based metabolomics approach, combining direct injection (DI) mass spectrometry (MS) and reverse-phase high-performance liquid chromatography (HPLC) tandem mass spectrometry (MS/MS). Chemical derivatization (using 3-nitrophenylhydrazine for organic acids or phenylisothiocynate for amines) was used in this method, followed by extraction, separation, and detection using multiple reaction monitoring (MRM) for metabolite identification and quantification.

Isotope-labeled ISTDs along with other ISTDs were used for accurate metabolite quantification. An Agilent 1290 series HPLC system (Agilent, Palo Alto, CA, USA) and an Agilent reversed-phase Zorbax Eclipse XDB C18 column (3.0 mm × 100 mm, 3.5 μm particle size, 80 Å pore size) were used for online LC-MS/MS with an AB SCIEX QTRAP^®^ 5500 mass spectrometer (AB SCIEX, Redwood City, CA, USA). The controlling software was Analyst^®^ 1.6.3. The mass spectrometer was set to positive/negative electrospray ionization with multiple reaction monitoring (MRM) mode. The raw data for metabolomics were profiled using Multi Quant^TM^ 3.0.3. [62]. Additional details regarding the method, derivatization strategy, separation protocol, MS methods, calibration, and metabolite quantification process were described previously [63].

### 4.9. Statistical Analysis

Statistical analysis was conducted by quantifying HPLC data of three independent experiments to assess the total adsorbed mols after 1 and 4 h of incubation for all types of nanoparticles (bare, MNP-APTES-GA, and Alb-MNPs). Statistical analysis was conducted using a two-way ANOVA, using Tukey’s multiple comparison tests. Calculated *p*-values were defined as *p* < 0.01 and *p* < 0.001.

## 5. Conclusions

The adsorption of UMs to albumin-coated MNPs was evaluated using model solutions of thirty-three UMs at concentrations similar to those observed for patients with kidney failure between HD sessions. The albumin solution concentration affected the thickness of the albumin coating formed on the MNPs (Figure 2), where higher solution concentrations (2 mg/mL) formed thicker films and had a lower zeta potential than the lower solution concentration (0.2 mg/mL). TGA and XPS data were used to confirm the higher amount of albumin coating with the increasing albumin solution concentration (Figure 3 and Figure 5). FTIR data were used to evaluate secondary structure changes for BSA and showed that the native structure was relatively retained for 2 mg/mL systems, and some dehydration occurred for thinner films formed at 0.2 mg/mL conditions (Figure 6). It was observed that metabolite binding was not related to solution concentration (Table 4). All types of particles adsorbed pyruvic acid and methylhistidine in substantially higher relative concentrations compared to the original solution concentration. The particles coated with glutaraldehyde and albumin of any concentration showed high selectivity for indoxyl sulfate. Both Alb-MNPs had higher selectivity for ethylphenyl sulphate than bare or glutaraldehyde-coated particles. Additionally, both types of Alb-MNPs showed that as the incubation time increased from 1 to 4 h, there was an increase in the adsorption of individual metabolites (except for pyruvic acid and kynurenine; Table 5). In contrast, bare and glutaraldehyde-coated particles exhibited the opposite trend. Unlike unmodified MNP surfaces, Alb-MNPs adsorbed PBUTs and non-PBUTs of middle and low molecular weight, like xanthosine, methylhistidine, and dimethylglycine. Notably, inconsistent adsorption was reported for indole-3-acetic acid on previously reported CD films, while its adsorption increased on average for all Alb-MNPs with time (Figure 9) [30]. Similar to other work on CD films, adsorption was substantially increased across all surfaces over time for hippuric acid, indoxyl sulfate, and p-cresol sulfate [30]. Moreover, Alb-MNPs exhibited a large amount of adsorption of indole acetic acid, indoxyl glucuronide, 3-deoxyglucosone, quinolinic acid, and kynurenine. CD films were shown to bind more PBUTs than albumin films, likely due to the sheer number of potential binding sites afforded by CD films, but Alb-MNPs showed a greater diversity in adsorbed species. Now that it has been shown that these systems bind UMs from model solutions, further research is needed to evaluate metabolite binding from complex solutions and the general hemocompatibility of these materials.

## Figures and Tables

**Table 1:** Composition of uremic toxin solution developed based on literature analysis of the blood of patients with kidney failure.

Uremic Toxin	Patient Concentration (Ave ± 1 SD, mg/L)	Experimental Concentration (mg/L)	Solution Composition (mol%)	Ref.
3-Deoxyglucosone	1.7 ± 1.0	1.7	0.131	[28]
4-Ethylphenyl sulfate	0.242 ± 0.044	0.25	0.015	[31]
Argininic acid	<0.077	0.077	0.005	[28]
Asymmetric dimethylarginine	0.385 ± 0.2884	0.385	0.024	[29]
Creatinine	136.0 ± 46.0	136	15.023	[28]
Dimethyl glycine	0.5768	0.59	0.071	[28]
Guanidinopropionic acid	0.288 ± 0.0183	0.29	0.028	[28]
Hippuric acid	247.0 ± 112	236	16.454	[28]
Homocysteine	8.1 ± 1.6	8.1	0.749	[28]
Hypoxanthine	2.0 ± 1.6	2	0.184	[28]
Indole acetic acid	2.03 ± 0.38	2.03	0.145	[29]
Indoxyl glucuronide	2.5 ± 0.3	2.5	0.101	[29]
Indoxyl sulfate	53.0 ± 91.5	53	3.106	[28]
Kynurenine	0.7~1.0	0.75	0.045	[29]
L-Asparagine	7.13 ± 3.7	7.13	0.674	[32]
L-Tyrosine	54.35 ± 16.3	54.35	3.747	[33]
Methylhistidine	0.08459 ± 0.05	0.085	0.006	[33]
Orotic acid	0.928 ± 0.489	0.928	0.074	[4]
p-Cresol sulfate	20.9 ± 12.2	20.9	1.387	[29]
Phenylacetic acid	467.2 ± 10.6	467.2	42.887	[29]
Phenylalanine	8.92 ± 1.81	9.25	0.7	[34]
p-Hydroxy hippuric acid	4.43 ± 2.79	4.25	0.272	[35]
p-Hydroxyphenyl acetic acid	2.43 ± 2.28	2.5	0.205	[35]
Putrescine	0.00942 ± 0.00759	0.00942	0.001	[29]
Pyruvic acid	11.7 ± 8.6	11.7	1.661	[36]
Quinolinic acid	0.0835	0.084	0.006	[26]
Spermidine	0.097 ± 0.045	0.096	0.008	[29]
Spermine	0.018 ± 0.0162	0.018	0.001	[28]
Trimethylamine n-oxide	7.49 ± 2.39	7.5	1.248	[37]
Uric acid	83 ± 13	83	6.169	[38]
Uridine	9.8 ± 11.4	9.8	0.501	[28]
Xanthine	1.5 ± 0.8	1.5	0.123	[28]
Xanthosine	96.6 ± 62.9	96.6	4.247	[39]

**Table 2:** XPS chemical composition of bare and subsequently added APTES, GA BSA (0.2), and BSA (2) nanoparticles. All component values are in atomic %.

Sample	MNPs	MNPs-APTES	MNPs-APTES-GA	MNPs-APTES-GA-BSA (0.2)	MNPs-APTES-GA-BSA (2)
Fe	18.75	15.28	10.97	5.94	1.78
C	33.79	33.00	45.14	56.54	74.18
O	47.46	46.79	36.88	27.21	18.11
N	-	2.01	3.29	8.22	5.47
Si	-	2.93	3.71	2.10	0.46

**Table 3:** The percentage of secondary structure content for pure BSA (i.e., in solution) and BSA incorporated into the coating on the MNP surface. Data represents the average ± 2 STD, n = 3.

Secondary Structure	Pure BSA	MNPs-APTES-GA-BSA (0.2)	MNPs-APTES-GA-BSA (2)
β-sheets/turns	10 ± 2	9 ± 2	17 ± 2
α-helix	65 ± 2	55 ± 2	60 ± 2
Random chains	5 ± 2	9 ± 2	7 ± 2
Extended chains/β-sheets	20 ± 2	21 ± 2	16 ± 2
Dehydrated β-turns	-	6 ± 2	

**Table 4:** Heat map illustrating binding specificity *.

	Bare MNPs		MNP-APTES-GA		MNP-APTES-GA-BSA		MNP-APTES-GA-BSA	
					(0.2 mg/mL)		(2 mg/mL)	
Uremic metabolites	1 h	4 h	1 h	4 h	1 h	4 h	1 h	4 h
3-Deoxyglucosone	0.30	0.31	0.32	0.20	0.23	0.24	0.19	0.36
4-Ethylphenyl Sulfate	1.11	0.78	0.99	1.01	1.26	1.24	0.80	1.27
Argininic Acid	-	-	-	-	-	-	-	-
Asymmetric Dimethylarginine	-	-	-	-	-	-	-	-
Creatinine	1.82	1.06	1.18	0.87	1.19	1.27	0.95	1.18
Dimethyl Glycine	0.59	0.40	0.50	0.29	0.41	0.59	0.29	0.42
Guanidinopropionic Acid	0.72	0.73	0.67	0.49	0.64	0.82	0.40	0.72
Hippuric Acid	1.09	0.88	0.99	0.66	0.84	1.03	0.60	0.85
Homocysteine	-	-	-	-	-	-	-	-
Hypoxanthine	1.34	0.87	1.13	0.73	1.09	1.22	0.94	1.14
Indole Acetic Acid	1.00	0.84	0.89	0.73	0.98	1.04	0.58	0.87
Indoxyl Glucuronide	0.76	0.53	0.69	0.47	0.63	0.65	0.39	0.60
Indoxyl Sulfate	1.07	0.76	1.20	1.46	1.60	1.73	1.01	1.69
Kynurenine	1.80	1.63	-	0.21	0.64	0.12	0.94	0.38
Methylhistidine	4.10	2.44	3.63	1.93	2.02	2.95	1.76	3.20
Orotic Acid	1.14	0.95	1.08	0.86	0.93	1.04	0.67	1.15
p-Cresol Sulfate	0.35	0.27	0.33	0.29	0.31	0.33	0.23	0.34
Phenylacetic Acid	-	-	-	-	-	-	-	-
Phenylalanine	1.14	0.58	0.78	0.53	0.76	0.99	0.52	0.82
p-Hydroxyhippuric Acid	0.81	0.76	0.82	0.60	0.74	0.89	0.50	0.77
Pyruvic Acid	24.14	35.17	31.50	38.70	32.84	29.00	39.75	31.73
Quinolinic Acid	1.16	0.77	1.09	0.74	0.99	1.05	0.64	1.02
Spermidine	-	-	-	-	-	-	-	-
Spermine	-	-	-	-	-	-	-	-
Trimethylamine N-Oxide	0.97	0.56	0.73	0.55	0.70	0.83	0.49	0.74
Uric Acid	0.84	0.70	0.83	0.55	0.67	0.82	0.56	0.79
Uridine	1.01	0.61	0.80	0.59	0.74	0.87	0.52	0.78
Xanthine	-	-	-	-	-	-	-	-
Xanthosine	0.48	0.45	0.41	0.38	0.38	0.41	0.26	0.52

* Table 4 displays a heat map of the binding specificity. The values were calculated with the ratio of the normalized, experimentally adsorbed mol% to its mol% in the original solution. Values greater than 1 (dark red) indicate enhanced adsorption relative to the compound’s initial concentration. Adsorption levels were further categorized as follows: 0.99 to 0.8 (medium red), 0.79 to 0.6 (light red), 0.59 to 0.4 (light blue), 0.39 to 0.2 (medium blue), and 0.19 to 0 (dark blue), representing approximately three-quarters, half, one-third, and less than one-fifth of the initial solution concentration, respectively. The “-” symbol denotes cases where calculations were not possible due to detection limits.

**Table 5:** Percent change in mol% from 1 to 4 h of incubation *.

Uremic Metabolites	Bare MNPs	MNP-APTES-GA	MNP-APTES-GA-BSA (0.2 mg/mL)	MNP-APTES-GA-BSA (2 mg/mL)	
3-Deoxyglucosone	1.7	−60.0	5.2	47.1	>30
4-Ethylphenyl Sulfate	−41.5	1.9	−1.5	37.3	10 to 29
Argininic Acid	-	-	-	-	−9 to 9
Asymmetric Dimethylarginine	-	-	-	-	−29 to −10
Creatinine	−71.9	−36.0	6.2	19.2	−49 to −30
Dimethyl Glycine	−48.5	−72.3	31.0	30.8	<−50
Guanidinopropionic Acid	0.5	−35.9	22.4	44.5	
Hippuric Acid	−24.2	−50.4	18.9	30.3	
Homocysteine	-	-	-	-	
Hypoxanthine	−53.5	−55.2	10.7	17.6	
Indole Acetic Acid	−19.6	−21.5	5.7	34.0	
Indoxyl Glucuronide	−44.7	−47.2	3.6	34.8	
Indoxyl Sulfate	−42.2	17.9	7.3	40.5	
Kynurenine	−10.6	100.0	−425.0	−146.1	
Methylhistidine	−67.6	−87.8	31.6	44.8	
Orotic Acid	−20.1	−25.4	10.9	41.9	
p-Cresol Sulfate	−30.0	−14.7	6.8	32.0	
Phenylacetic Acid	-	-	-	-	
Phenylalanine	−96.0	−46.4	23.6	36.3	
p-Hydroxyhippuric Acid	−7.4	−36.8	16.7	35.3	
Pyruvic Acid	31.4	18.6	−13.2	−25.3	
Quinolinic Acid	−51.5	−48.5	5.9	36.8	
Spermidine	-	-	-	-	
Spermine	-	-	-	-	
Trimethylamine N-Oxide	−73.3	−33.2	16.4	33.6	
Uric Acid	−20.3	−50.8	18.9	29.3	
Uridine	−65.8	−35.0	14.4	33.3	
Xanthine	-	-	-	-	
Xanthosine	−5.9	−7.2	6.3	48.8	

* Red represents an increase, blue indicates a decrease, and white signifies minimal change.

## Data Availability

The data used to support the findings of this study are included in the article.

## Abbreviations

The following abbreviations are used in this manuscript:

PBUT	Protein-bound uremic toxin
CKD	Chronic kidney disease
UM	Uremic metabolite
HD	Hemodialysis
MNP	Magnetic nanoparticle
p(MPC-co-PMβCD)	(Methacryloyloxy)ethyl phosphorylcholine-co-β-cyclodextrin
TGA	Thermogravimetric analysis
XPS	X-ray photoelectron spectroscopy
FTIR	Fourier-transform infrared spectroscopy
TEM	Transmission electron microscopy
FSD	Fourier self-deconvolution
PBS	Phosphate-buffered saline
HPLC	High-performance liquid chromatography
DI	Direct injection
MS	Mass spectrometry
MS/MS	Tandem mass spectrometry
MRM	Multiple reaction monitoring
BSA	Bovine serum albumin
Alb-MNP	Albumin-modified MNPs
(-NH2-)	Amide-I
(-NH-)	Amide-II
CD	Cyclodextrin
